# LCS-1 inhibition of superoxide dismutase 1 induces ROS-dependent death of glioma cells and degradates PARP and BRCA1

**DOI:** 10.3389/fonc.2022.937444

**Published:** 2022-08-01

**Authors:** Min Ling, Qing Liu, Yufei Wang, Xueting Liu, Manli Jiang, Jinyue Hu

**Affiliations:** ^1^ Department of Neurosurgery, Xiangya Hospital, Central South University, Changsha, China; ^2^ Department of Clinical Laboratory, Changsha Central Hospital, Hengyang Medical School, University of South China, Changsha, China; ^3^ Medical Research Center, Changsha Central Hospital, Hengyang Medical School, University of South China, Changsha, China

**Keywords:** glioma, LCS-1, SOD1, ROS, PARP, BRCA1, cell death

## Abstract

Gliomas are characterized by high morbidity and mortality, and have only slightly increased survival with recent considerable improvements for treatment. An innovative therapeutic strategy had been developed *via* inducing ROS-dependent cell death by targeting antioxidant proteins. In this study, we found that glioma tissues expressed high levels of superoxide dismutase 1 (SOD1). The expression of SOD1 was upregulated in glioma grade III and V tissues compared with that in normal brain tissues or glioma grade I tissues. U251 and U87 glioma cells expressed high levels of SOD1, low levels of SOD2 and very low levels of SOD3. LCS-1, an inhibitor of SOD1, increased the expression SOD1 at both mRNA and protein levels slightly but significantly. As expected, LCS-1 caused ROS production in a dose- and time-dependent manner. SOD1 inhibition also induced the gene expression of HO-1, GCLC, GCLM and NQO1 which are targeting genes of nuclear factor erythroid 2-related factor 2, suggesting the activation of ROS signal pathway. Importantly, LCS-1 induced death of U251 and U87 cells dose- and time-dependently. The cell death was reversed by the pretreatment of cells with ROS scavenges NAC or GSH. Furthermore, LCS-1 decreased the growth of xenograft tumors formed by U87 glioma cells in nude mice. Mechanistically, the inhibition of P53, caspases did not reverse LCS-1-induced cell death, indicating the failure of these molecules involving in cell death. Moreover, we found that LCS-1 treatment induced the degradation of both PARP and BRCA1 simultaneously, suggesting that LCS-1-induced cell death may be associated with the failure of DNA damage repair. Taking together, these results suggest that the degradation of both PARP and BRCA1 may contribute to cell death induced by SOD1 inhibition, and SOD1 may be a target for glioma therapy.

## Introduction

Owing to the localization and the often locally invasive growth, central nervous system tumors are characterized by high morbidity and mortality (1). Gliomas are the most common types of primary brain tumors, accounting for almost 30% of all primary brain tumors, and 80% of all malignant types, and are responsible for the majority of deaths from primary brain tumors (1). Conventional treatment strategies of gliomas provide a gross total removal of tumors, which are associated with several cycles of radiotherapy and chemotherapy (1). Though considerable improvements in terms of surgical approaches including operative microscopes and image guided surgery have been reached, patients have only slightly increased survival (1). So optimal therapeutic strategy is required to improve the therapeutic effects and increase patient survival.

Reactive oxygen species (ROS) are oxygen-containing free radicals which are derived from the partial reduction of oxygen (2). ROS have dual roles in cell metabolism. At low to moderate levels, ROS act as signal transducers to activate cell proliferation, migration, invasion, and angiogenesis. In contrast, high levels of ROS cause damage to proteins, nucleic acids, lipids, membranes, and organelles, leading to cell death (3). Typically, cancer cells exhibit high levels of ROS compared with normal cells as a result of an imbalance between oxidants and antioxidants (3). Anticancer therapeutic strategies have been developed by manipulating ROS levels *via* inducing more oxidants and/or targeting antioxidants (3). By modulating ROS, a number of natural or synthesized compounds have been used for cancer therapy (4–9).

Superoxide Dismutases (SOD) are highly conserved enzymes, which play fundamental roles in protecting cells from oxidative stress by catalyzing the dismutation of the superoxide radical (10). There are three forms of SOD that incorporate different covalently bound substances (Mn, Zn, Cu, Fe), and inactivate both intra- and extra-cellular superoxides (10). SOD1 (Cu/Zn SOD), which contains copper and zinc, localizes in the cytoplasm, nuclei, lysosomes and peroxisomes, and also in mitochondrial intermembrane space (10). SOD2 (Mn SOD) contains manganese and is predominantly observed in the mitochondrial matrix (10). The third one, SOD3 (Cu/Zn extracellular SOD), also contains copper and zinc but is secreted to the extracellular space (10). SOD1 is a 15.9 kDa homodimer which is held by hydrophobic contacts that reduce solvent accessibility and increase its stability. Each monomer contains a copper and a zinc ion, which together have either a structural or catalytic function. Beside its enzymatic activity to dismutae superoxide radical, SOD1 translocates nuclei as a transcription factor to regulate the expression of oxidative resistance and repair genes in response to high levels of hydrogen peroxide (11).

In cancer cells, the dysfunction of SOD1 causes ROS-dependent cell damage which should benefit for cancer therapy. Early, SOD1 inhibitor ATN-224 has been reported to attenuate angiogenesis and tumor cell proliferation (12). ATN-224 has also been reported to induce cell death in various NSCLC cells, including those harboring KRAS mutations (13). Another samll molecular LCS-1 (lung cancer screen 1, 4,5-Dichloro-2-(3-methylphenyl)-3(2H)-pyridazinone) is screened as an inhibitor of SOD1 and inhibits the growth of lung adenocarcinoma cell lines (14), and has been reported to induce death of colorectal cancer cells and breast cancer cells (15–17). However, the effect of SOD1 inhibition on glioma therapy is not understood. Especially, the detailed mechanism of cell death induced by SOD1 inhibition remains elusive in cancer cells.

In this study, we found that clinical glioma expressed increased SOD1. LCS-1 inhibition of SOD1 induced ROS-dependent cell death in glioma cells, and decreased glioma growth *in vivo*. Mechanistically, LCS-1-induced cell death was not associated with P53 and caspase. But it may be associated with PARP and BRCA1, because PARP inhibitors induce anti-cancer effect in BRCA1-mutant cancer types, and LCS-1 induced the degradation of both PARP and BRCA1 simultaneously.

## Materials and methods

### Cell lines

Hunan glioma cell line U87 was purchased from ATCC (Manassas, VA, USA). U251 is a human glioma cell line as well (18). Cells were grown in DMEM, containing 10% FCS, 100 units/ml penicillin, and 100 mg/ml streptomycin. All cells were cultured in a humidified atmosphere with 5% CO_2_ at 37°C.

### Animals

Female BALB/c nude mice (6–8 weeks old) were purchased from SLAC Laboratory Animal Center (Shanghai, China). All animal studies were carried out in accordance with the Guidelines for the Care and Use of Laboratory Animals issued by the National Institutes of Health and approved by the Animal Ethics Committee of the Changsha Central Hospital, University of South China (No. CCH-AEC-2020-02). Animals were maintained with standard rodent chow and free access to water under controlled conditions with a 12-h light and 12-h dark cycle and a temperature of 24 ± 2°C.

### Reagents

Mouse monoclonal anti-human SOD1 (sc-101523), SOD2 (sc-137254), BRCA1 (sc-6954) and BRCA2 (sc-518154) were purchased from Santa Cruz Biotechnology (Santa Cruz, CA, USA). Rabbit anti-human caspase 3 (9662), PARP (9532), HO-1 (5853) antibodies were purchased from Cell Signaling Technology (Beverly, MA, USA). ROS scavengers N-acetyl cysteine (NAC, ST1546) and reduced glutathione (GSH, S0073), DCF ROS assay kit (S0033), pan-caspase inhibitor Z-vad-FMK (C1202), and a mouse monoclonal anti-human GAPDH antibody (AF5009) were purchased from Beyotime (Shanghai, China). PARP inhibitor PJ34 (3255), were purchased from Tocris (Ellisville, MO, USA). SOD1 inhibitor LCS-1 (567417) was purchased from Merck (Darmstadt, Germany). Recombinant human EGF (AF-100-15), IL-6 (200-06) were purchased from PeproTech (Rocky Hill, NJ, USA).

### Immunoblot

1 – 2 × 10^6^ cells were lysed in 200 ml lysis buffer (20 mM Tris, pH 7.5, 150 mM NaCl, 1% Triton X-100, 1 mM EDTA, 1 mM β-glycerophosphate, 1 mM sodium pyrophosphate, 1 mM Na_3_VO_4_, 1 mg/ml leupeptin). The cell lysate was centrifuged at 12,000 g for 5 min at 4°C. Proteins were electrophoresed on 8-10% SDS-PAGE gels, and transferred onto Immobilon P membranes (Millipore, Billerica, MA, USA). The membranes were blocked by incubation in 3% nonfat dry milk at room temperature for 1 h and then incubated with primary antibodies in PBS containing 0.01% Tween 20 at 4°C overnight. After incubation with a horseradish peroxidase-conjugated secondary antibody, the protein bands were detected with SuperSignal chemiluminescent substrate-stable peroxide solution (Pierce Rockford, IL, USA) and BIOMAX-MR film (Eastman Kodak Co., Rochester, NY, USA). When necessary, the membranes were stripped with Restore Western Blot Stripping Buffer (Pierce) and re-probed with antibodies against various cellular proteins.

### Quantitative reverse transcriptional-polymerase chain reaction (qRT-PCR)

The qRT-PCR was performed as described by Sun et al. (19). Briefly, total RNA was extracted from 1 - 2 × 10^6^ cells by use of TRIzol (Invitrogen, Carlsbad, CA, USA) as described by the manufacturer. mRNA was reverse transcribed with RevertAid (MBI Fermentas, Burlington Ontario, Canada) at 42°C for 60 min. cDNA was amplified by use of TaqMan Universal PCR master mix (Roche Applied Science) and a LightCycle 96 detection system (Roche Applied Science). The amplification of the target genes was normalized by use of the amplification levels of glyceraldehyde-3-phosphate dehydrogenase (GAPDH) as an endogenous control. The efficiency of the PCR was tested by amplification of the target from serially diluted cDNA generated from the reverse transcription of a stock set of human RNA. The data analysis and calculations were performed using the 2^−ΔΔ^ *^CT^* comparative method, as described by the manufacturer. Gene expression is shown as the fold induction of a gene measured in LCS-1-treated samples relative to samples cultured with medium. The forward and reverse primer pairs are listed (5' to 3') as follows:

BCL2-S: CGTTTGGCAGTGCAATGGT,

BCL2-A: TTCTTGATTGAGCGAGCCTT;

GAPDH-S, AATCCCATCACCATCTTCCA,

GAPDH-A, CCTGCTTCACCACCTTCTTG;

GCLC-S: ATCCTCCAGTTCCTGCACAT,

GCLC-A: TTTTCGCATGTTGGCCTCAA;

GCLM-S: TCCTTGGAGCATTTACAGCC,

GCLM-A: AGAGCTTCTTGGAAACTTGCT;

HO-1-S: CCAGTCTTCGCCCCTGTC,

HO-1-A: GGGCTTTCTGGGCAATCTTT;

MDM2-S: TTCGTGAGAATTGGCTTCC,

MDM2-A: GGCAGGGCTTATTCCTTTTCT;

Noxa-S: CCAAACTCTTCTGCTCAGGAA,

Noxa-A: ATCACAGGTCATCTCCCTTCA;

NQO1-S: GTCGGACCTCTATGCCATGA,

NQO1-A: GGGTCCTTTGTCATACATGGC;

SOD1-S: AGGGCATCATCAATTTCGAGC,

SOD1-A: TGATGCAATGGTCTCCTGAG;

SOD2-S: ACATCAACGCGCAGATCATG,

SOD2-A: CAACAGATGCAGCCGTCAG;

SOD3-S: CCACCATCCTTCCATCCTGA,

SOD3-A: GAAACAGCTGAAGACGCGG;

### DCF staining assay for measurement of ROS

Intracellular ROS levels were measured by DCF ROS assay according to the manufacturers’ standard protocols. Briefly, 1 – 2 × 10^6^ cells were cultured in FCS-free medium with 10 μM dichlorofluorescein diacetate (DCFH-DA) at 37°C for 30 min, and then washed with FCS-free medium trice, followed by the treatment with various reagents described in figure legends. ROS in the cells causes the oxidation of DCFH-DA, yielding the fluorescent product 2′,7′-dichlorofluorescein (DCF). The fluorescence of DCF was measured using a FACScan (BD Bioscience, San Jose, CA). For each analysis, 10,000 events were recorded.

### Flow cytometric analysis

Cell death was detected by propidium iodide (PI)/fluorescein isothiocyanate (FITC)-annexin V staining. Briefly, 1 - 2 × 10^6^ cells were washed twice with PBS and then labeled with FITC-annexin V and PI in binding buffer according to manufacturer’s instructions. The fluorescence signals were detected on a FACScan (BD Bioscience, San Jose, CA). The log of FITC-annexin V–fluorescence was displayed on the x-axis, and the log of PI fluorescence was displayed on the y-axis. For each analysis, 10,000 events were recorded.

For protein detection, cells were cultured in 6 well plates for 24 h, and harvested and washed with fluorescence-activated cell sorting buffer (5 mmol/L EDTA, 0.1% NaN 3, and 1% FCS in Dulbecco’s PBS). After incubation with an antibody against human SOD1 or SOD2 for 30 min on ice, the cells were stained with a FITC-labeled secondary antibody and protein expression was examined by flow cytometry (BD Bioscience, San Jose, CA).

### Tissue microarray and immunohistochemistry

For immunohistochemistry, a tissue microarray (78 samples) was purchased from Bioaitech company (F1081301, Xian, China), which contained four samples of brain normal tissue, 10 samples of glioma adjacent tissue, 64 samples of glioma. The use of the human tissue microarrays was approved by the ethics committee of Changsha Central Hospital, University of South China. Immunohistochemistry was performed to detect SOD1 expression as described in a previous study (20). Positive staining was evaluated in random four fields (100 cells) under microscope at 400× magnification. The staining intensity was scored as follows: 0 = no expression, 1 + = weak expression, 2 + = moderate expression, 3 + = strong expression, and 4 + = very strong expression. The final scores were expressed as immunohistochemical staining scores (IHC scores) obtained by multiplying the percentage of positive cells with the staining intensity (21).

### Implantation of cervical cancer cells in nude mice

Female BALB/c nude mice (6 – 8 weeks old) from SLAC Laboratory Animal Center (Shanghai, China) were used in all experiments. 1× 10^7^ U87 glioma cells in 200 μl PBS were implanted by s.c. injection into the right flanks of the mice. At day 15 after initial implantation, 10 mice were divided into two groups. In experimental group, five mice were injected i.p. with LCS-1 (400 nmol per mouse) every two days for 14 times beginning at day 15. Five mice were injected with vehicle as control. The growth of implanted tumors was examined every two days. Tumor sizes were calculated by the formula LW^2^/2, where L is the length of the tumors in centimeters and W is the width of the tumors in centimeters. At day 45, all mice were euthanized, and the weight of tumors and mouse bodies was measured. Animal care was provided in accordance with the Guide for the Care and Use of Laboratory Animals.

### Statistical analysis

All experiments were performed at least three times, and the representative results were shown. The results were expressed as the mean ± S.D. Differences between groups were examined for statistical significance using two tailed Student’s *t* test, and *P* values equal to or < 0.05 were considered statistically significant (n = 3 for each qRT-PCR and ELISA test).

## Results

### The expression of SOD1 was up-regulated in glioma tissues

**Figure 1:**
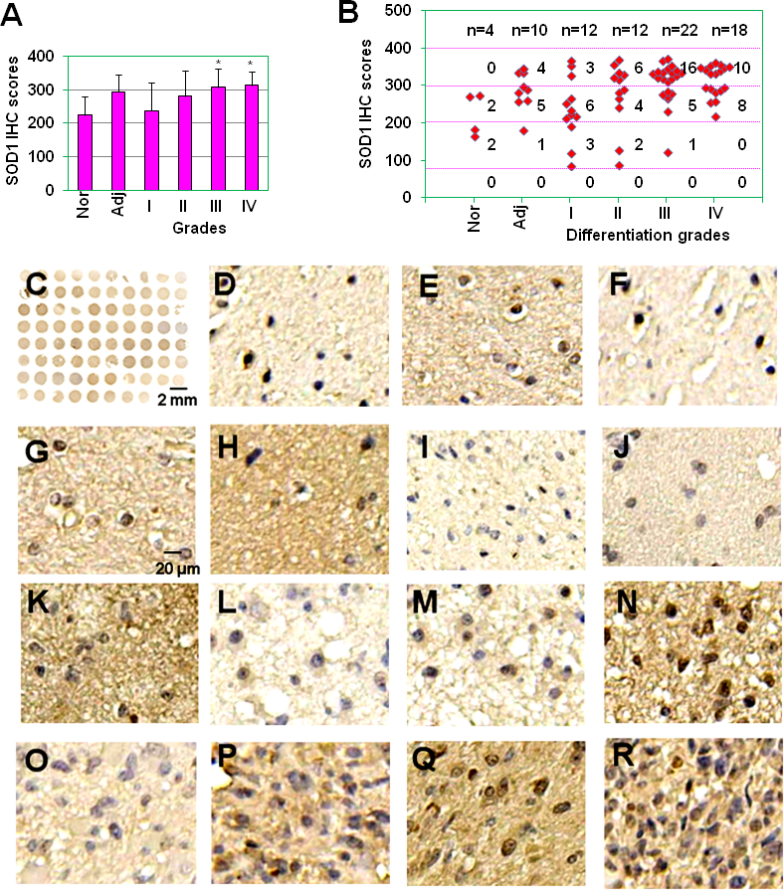
The expression of SOD1 in glioma tissues. **(A)** SOD1 IHC scores of normal brain tissues, tumor adjacent tissues and glioma grade I, II, III and IV tissues. * *P* < 0.05 compared with the normal brain tissue (Nor) or glioma grade I tissues. **(B)** Staining intensity of glioma with different histopathological types. Staining intensity was scored using a four-tier scale and defined as follows: negative staining (0-80); weak staining (80-200); moderate staining (200-300); strong staining (300-400). Nor, normal brain tissue; Adj, glioma adjacent tissues; I, well differentiated glioma; II, moderately differentiated glioma; III, poorly differentiated glioma; and IV, non-differentiated glioma. **(C)** SOD1 IHC staining for full glioma microarray. **(D-E)** Representative SOD1 weak **(D)**, moderate staining **(E)** in normal brain tissues. **(F-H)** Representative SOD1 weak **(F)**, moderate **(G)**, strong staining **(H)** in glioma adjacent tissues. **(I-K)** Representative SOD1 weak **(I)**, moderate **(J)**, strong staining **(K)** in glioma grade I tissues. **(L, M)** Representative SOD1 weak **(L)**, moderate **(M)**, strong staining **(N)** in glioma grade II tissues. **(O-Q)** Representative SOD1 weak **(O)**, moderate **(P)**, strong staining **(Q)** in glioma grade III tissues. **(R)** Representative SOD1 strong staining in glioma grade IV tissues.

Emerging evidences indicate that SOD1 is overexpressed in cancers and is essential to maintain cellular redox homeostasis under the condition with excessive ROS derived from the aberrant metabolism (22, 23). However, the expression of SOD1 in gliomas is still unknown. In this study, SOD1 expression was first detected in glioma tissue microassay (78 samples) which included four samples of brain normal tissues, 10 samples of glioma adjacent tissues, 12 samples of glioma grade I tissues, 12 samples of glioma grade II tissues, 22 samples of glioma grade III tissues, and 18 samples of glioma grade IV tissues (Bioaitech). The expression levels of SOD1 were evaluated by use of immunohistochemical (IHC) scores. The results showed that SOD1 IHC scores were 224 ± 57, 294 ± 51, 238 ± 85, 281 ± 78, 308 ± 55, 314 ± 42 for normal tissues, glioma adjacent tissues, glioma grade I, II, III and IV tissues respectively (**Figure 1A**). Statistical results showed that the expression scores of SOD1 in glioma grade III and IV tissues were significantly higher than that in brain normal tissues and glioma grade I tissues (*P* < 0.05) (**Figure 1A**). In brain normal tissues, non-sample was SOD1 negative staining (scores: 0-80), two samples were SOD1 weak staining (scores: 81-200), two samples were SOD1 moderate staining (scores: 201-300) and non-sample was SOD1 strong staining (301–400) (**Figure 1B**). In glioma adjacent tissues, non-sample was SOD1 negative staining, one sample was SOD1 weak staining, five samples were SOD1 moderate staining, and four samples were SOD1 strong staining (**Figure 1B**). In glioma grade I tissues, non-sample was SOD1 negative staining, three samples were SOD1 weak staining, six samples were SOD1 moderate staining, and three samples were SOD1 strong staining (**Figure 1B**). In glioma grade II tissues, non-sample was SOD1 negative staining, two samples were SOD1 weak staining, four samples were SOD1 moderate staining and 6 samples were SOD1 strong staining (**Figure 1B**). In glioma grade III tissues, non-sample was SOD1 negative staining, one sample was SOD1 weak staining, five samples were SOD1 moderate staining and 16 samples were SOD1 strong staining (**Figure 1B**). In glioma grade IV tissues, non-sample was SOD1 negative staining, non-sample was SOD1 weak staining, 8 samples were SOD1 moderate staining and 10 samples were SOD1 strong staining (**Figure 1B**). **Figure 1C** showed the immunohistochemical (IHC) staining of SOD1 in the full tissue microarray. **Figures 1D, E** showed the representative SOD1 weak, moderate staining in normal brain tissues. **Figures 1F-H** showed the representative SOD1 weak, moderate, strong staining in glioma adjacent tissues. **Figures 1I-K** showed the representative SOD1 weak, moderate, strong staining in glioma grade I tissues. **Figures 1L-N** showed the representative SOD1 weak, moderate, strong staining in glioma grade II tissues. **Figures 1O-Q** showed the representative SOD1 weak, moderate, strong staining in glioma grade III tissues. **Figure 1R** showed the representative SOD1 strong staining in glioma grade IV tissues. These results indicate that SOD1 expression is increased in clinical gliomas.

### The expression of SOD1 in glioma cell lines

**Figure 2:**
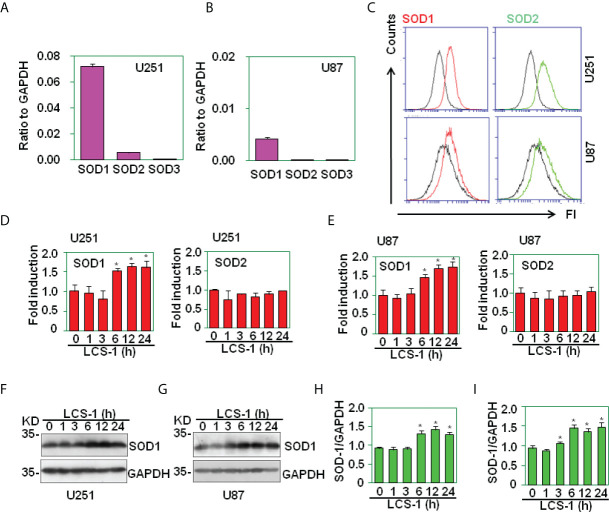
The expression of SOD1, SOD2 and SOD3 in glioma cell lines. **(A)** qRT-PCR analysis of SOD1, SOD2 and SOD3 mRNA levels in U251 cells. **(B)** qRT-PCR analysis of SOD1, SOD2 and SOD3 mRNA levels in U87 cells. **(C)** FACS analysis of SOD1 and SOD2 protein levels in U251 and U87 cells. **(D)** qRT-PCR analysis of SOD1 and 2 mRNA levels in U251 cells treated with 10 µM LCS-1 for the indicated time periods. * *P* < 0.05 compared with the medium groups. **(E)** qRT-PCR analysis of SOD1, SOD2 and SOD3 mRNA levels in U87 cells treated with 10 µM LCS-1 for the indicated time periods. * *P* < 0.05 compared with the medium groups. **(F, G)** Western blot analysis of the protein levels of SOD1 in U251 **(F)** and U87 **(G)** cells. **(H, I)** The quantitative data from F **(H)** and G **(I)** respectively. * *P* < 0.05 compared with the medium groups.

Before the testing of SOD1 inhibitor on cell survival in glioma, the expression of SOD1, SOD2 and SOD3 in U251 and U87 glioma cell lines was measured. qRT-PCR results showed that these two glioma cell lines expressed higher levels of SOD1, lower levels of SOD2, and very low levels of SOD3 (**Figures 2A, B** **)**. FACS results showed that U251 and U87 cells expressed SOD1 and SOD2 proteins (**Figure 2C**). When SOD1 was inhibited by LCS-1, qRT-PCR results showed that SOD1 mRNA levels were slightly but significantly up-regulated (**Figures 2D, E** **)**. Meanwhile, western blot results showed that LCS-1 treatment of U251 and U87 cells increased the protein levels slightly but significantly (**Figures 2F-I**). These results indicate that glioma cells expressed SOD1 and SOD2, and LCS-1 up-regulated SOD1.

### LCS-1 mediates ROS production

**Figure 3:**
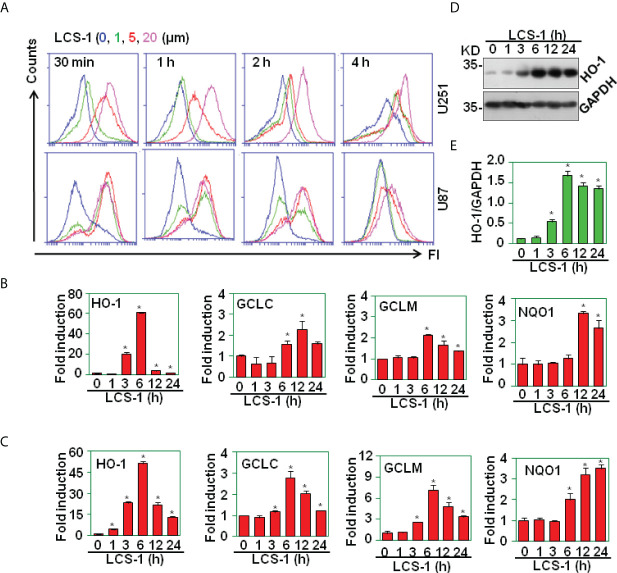
LCS-1 induces the production of ROS. **(A)** DCF staining analysis of ROS levels in U251 and U87 cells treated with the indicated doses of LCS-1 for the indicated time periods. **(B)** qRT-PCR analysis of the mRNA levels of NRF2-targeted genes in U251 cells treated with 10 µM LCS-1 for the indicated time periods. * *P* < 0.05 compared with the control groups. **(C)** qRT-PCR analysis of the mRNA levels of NRF2-targeted genes in U87 cells treated with 10 µM LCS-1 for the indicated time periods. * *P* < 0.05 compared with the control groups. **(D)** Western blot analysis of the protein levels of HO-1 in U87 cells treated with 10 μM LCS-1 for the indicated time periods. **(E)** Quantitative data from **(D)** * *P* < 0.05 compared with the medium groups.

The inhibition of SOD1 elicits the accumulation of ROS (22). To test the effect of SOD1 inhibitor LCS-1 on the production of ROS in U251 and U87 cells, DCF staining assay was used to detect the ROS levels in cells treated with LCS-1. FACS results showed that LCS-1 up-regulated ROS levels in a dose- and time-dependent manner (**Figure 3A**), suggest that SOD1 inhibition induced ROS production. To further test the activity of ROS induced by LCS-1, qRT-PCR was used to measure the expression of the targeting genes regulated by ROS pathways. The results showed that the mRNA levels of heme oxygenase-1 (HO-1), γ-glutamyl cysteine ligase modulatory and catalytic subunits GCLM and GCLC, NAD(P)H dehydrogenase quinone 1 (NQO1) were significantly increased in a dose- and time-dependent manner (**Figures 3B, C** **)**. Meanwhile, western blot results showed that the treatment of U87 cells with LCS-1 significantly up-regulated the protein levels of HO-1 in U87 cells (**Figures 3D, E** **)**. These results indicate that LCS-1 inhibition of SOD1 induces the production of ROS, and activates the ROS signaling pathways in glioma cell lines.

### SOD1 inhibitor LCS-1 induces cell death

**Figure 4:**
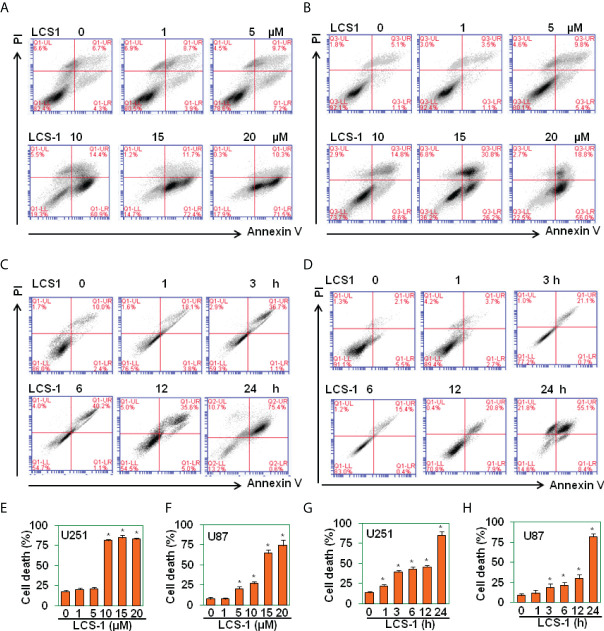
LCS-1 induces cell death in glioma cells. **(A-B)** PI/FITC-Annexin V staining of death of U251 **(A)** and U87 **(B)** cells treated with the indicated doses of LCS-1 for 24 h **(C, D)** PI/FITC-Annexin V staining of death of U251 **(C)** and U87 **(D)** cells treated with 20 µM LCS-1 for the indicated time periods. **(E-H)** Quantitative data from **A (E), B (F), C (G)** and **D (H)** respectively. * *P* < 0.05 compared with the control groups.

It is reported that SOD1 inhibitor LD100 promotes cancer cell apoptosis *via* regulating ROS signal pathway (23). In this study, SOD1 inhibitor LCS-1 induced ROS production, activated ROS signal pathway in glioma cells, indicating that LCS-1 may induce cell death in gliomas. By use of PI/FITC-Annexin V staining and FACS, we detected the effect of LCS-1 on the cell survival in U251 and U87 cells. The results showed that LCS-1 significantly induced cell death in both U251 and U87 cells in a dose- and time-dependent manner (**Figures 4A-H**). These results suggest that SOD1 inhibitor LCS-1 is an effective chemical for the induction of cell death in glioma cells.

### ROS scavengers reverse LCS-1-induced cell death

**Figure 5:**
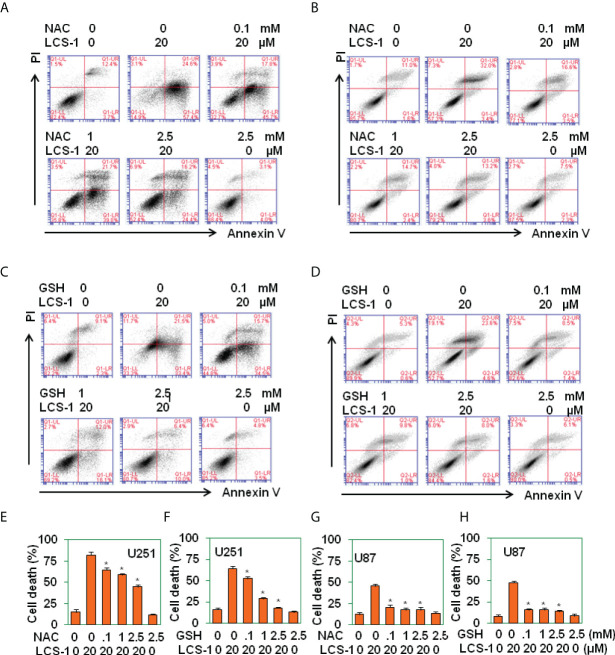
ROS scavenges reverse LCS-1-induces cell death. **(A-D)** PI/FITC-Annexin V staining of death of U251glioma cells pretreated with the indicated doses of NAC **(A)**, or GSH **(B)** for 1 h and re-treated with 20 µM LCS-1 for 24 h, and U87 cells pretreated with indicated doses of NAC **(C)**, or GSH **(D)** for 1h and retreated with 20 µM LCS-1 for 24 h **(E-H)** Quantitative data from **A (E), B (F), C (G)** and **D (H)** respectively. * *P* < 0.05 compared with LCS-1-treated alone groups.

To determine that LCS-1-induced cell death is related to the production of ROS, U251 and U87 cells were pretreated with ROS scavengers N-acetyl-L-cysteine (NAC) and reduced glutathione (GSH), and retreated with LCS-1, then cell death was measured. The results showed that in both U251 and U87 cells, NAC and GSH significantly reversed the cell death induced by LCS-1 in a dose-dependent manner (**Figures 5A-H**). These results indicate that LCS-1-induced cell death is ROS-dependent.

### LCS-1 inhibits glioma growth in nude mice model

**Figure 6:**
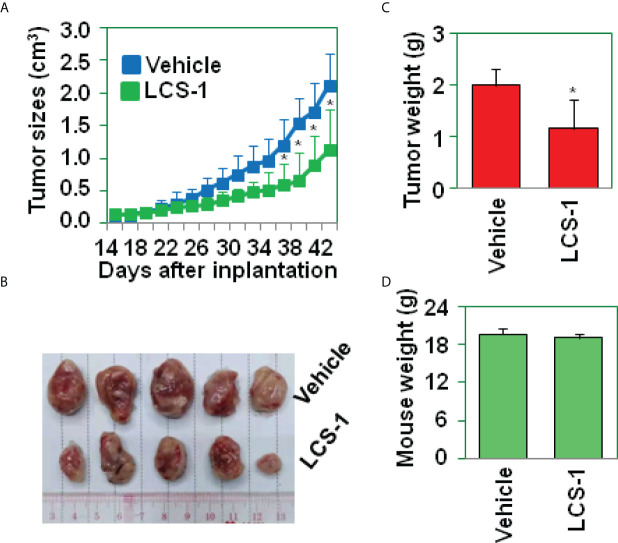
LCS-1 decreases tumor growth in nude mice. **(A)** Tumor growth curve from nude mice implanted with U87 cells and treated with or without LCS-1. * *P* < 0.05 compared with the vehicle groups. **(B)** Tumors from nude mice implanted with U87 cells and treated with or without LCS-1. **(C)** The weights of tumors from nude mice implanted with U87 cells and treated with or without LCS-1. * *P* < 0.05 compared with the vehicle group. **(D)** The weights of mice implanted with U87 cells and treated with or without LCS-1.

To determine the effect of LCS-1 on glioma growth *in vivo*, U87 glioma cells were implanted s.c. into the flanks of nude mice (n = 10). At day 15 after implantation, the mice (n = 5) in the experimental group were treated with LCS-1, and the mice (n = 5) in the control group were injected with vehicle. Tumors in LCS-1-treated mice grew more slowly than those in control mice (**Figure 6A**), as shown by the photographs in **Figure 6B**, as well as the weight of tumors shown in **Figure 6C**. However, there was no difference in the mouse body weight between LCS-1-treated and control groups (**Figure 6D**). These results suggest that LCS-1 inhibits glioma growth *in vivo* without causing side effects in mice.

### LCS-1-induced cell death is P53-independent

**Figure 7:**
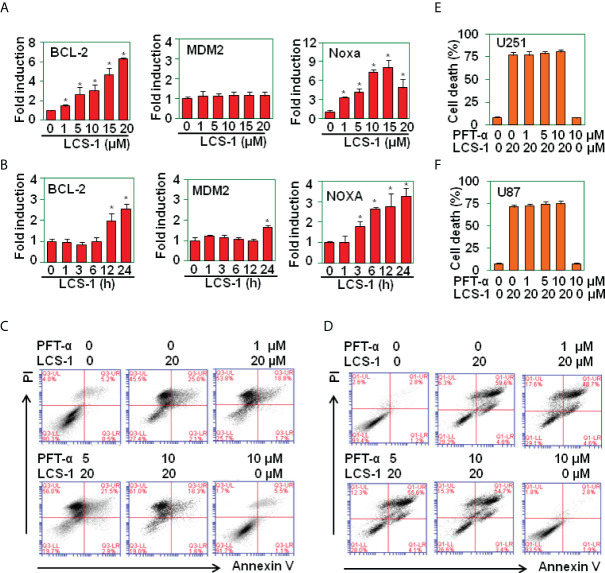
LCS-1-induced cell death is P53 in-dependent. **(A)** qRT-PCR analysis of the mRNA levels of P53-targeted genes in U251 cells treated with the indicated doses of LCS-1 for 12 h. * *P* < 0.05 compared with the medium groups. **(B)** qRT-PCR analysis of the mRNA levels of P53-targeted genes in U87 cells treated with 10 μM LCS-1 for the indicated time periods. * *P* < 0.05 compared with the medium groups. **(C, D)** PI/FITC-Annexin V staining of death of U251 **(C)** and U87 **(D)** cells pretreated with the indicated doses of P53 inhibitor Pifithrin-α (PFT-α) for 1 h and retreated with 20 μM LCS-1 for 24 h. **(E, F)** Quantitative data from **C (E)** and **D (F)**. * *P* < 0.05 compared with LCS-1-treated alone groups.

It has been reported that ROS activates P53 signaling pathway (24–26). To determine whether LCS-1-induced cell death is associated with P53 activation, P53-targeted genes including Bcl-2, MDM2, Noxa were measured by qRT-PCR. The results showed that the treatment of U87 cells with LCS-1 did not down-regulate the mRNA levels of Bcl-2, only slightly up-regulated the mRNA of MDM2 and Noxa (**Figures 7A, B** **)**, indicating that P53 did not involve in LCS-1-induced cell death. Furthermore, U87 cells were pretreated with P53 inhibitor Pifithrin-α (PFT-α), and retreated with LCS-1, then cell death was measured. The results showed that Pifithrin-α did not reverse LCS-1-induced cell death (**Figures 7C-F**). These results indicate that LCS-1-induced cell death is P53-independent.

### LCS-1-induced cell death is caspase-independent

**Figure 8:**
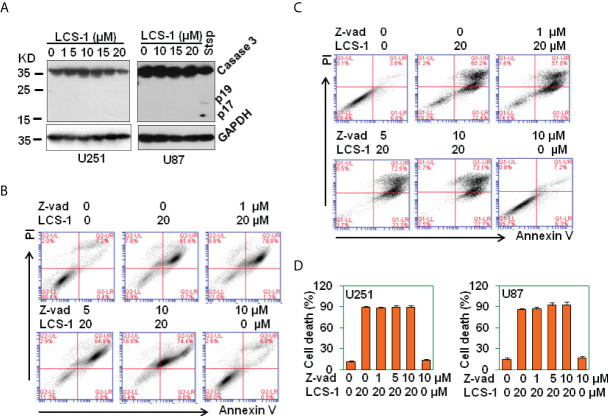
LCS-1-induced cell death is Caspase in-dependent. **(A)** Western blot analysis of caspase 3 activation in U251 and U87 cells treated with the indicated doses of LCS-1 for 24 h. U87 cells treated with 1 μM Staurosporine (Stsp) for 24 h as positive controls. GAPDH protein levels were measured as loading controls. **(B)** PI/FITC-Annexin V staining of death of U251 glioma cells pretreated with the indicated doses of pan-caspase inhibitor Z-vad-FMK (Z-vad) for 1 h, and retreated with 20 μM LCS-1 for 24 h **(C)** PI/FITC-Annexin V staining of death of U87 glioma cells pretreated with the indicated doses of pan-caspase inhibitor Z-vad-FMK (Z-vad) for 1 h. and retreated with 20 μM LCS-1 for 24 h **(D)** Quantitative data from B and C respectively.

It has been reported that ROS induces caspase-dependent cell death in breast cancer cells and in hepatocellular carcinoma (27, 28). To determine where LCS-1-induced cell death is associated with caspase activation, western blot was used to measure the activation of caspase 3. The results showed that LCS-1 treatment of U251 and U87 cells did not activate caspase 3, the cleaved fragments p19 and p17 were not observed (**Figure 8A**). As a control, staurosprine induced the activation of caspase 3, cleaved p19 and p17 were detected by western blot (**Figure 8A**). Meanwhile, when U251 and U87 cells were pretreated with caspase paninhibitor Z-vad-FMK, and then retreated with LCS-1, LCS-1-induced cell death was not reversed (**Figures 8B-D**). These results suggest that LCS-1-induced cell death is caspase-independent.

### LCS-1 induces degradation of PARP and BRCA1

**Figure 9:**
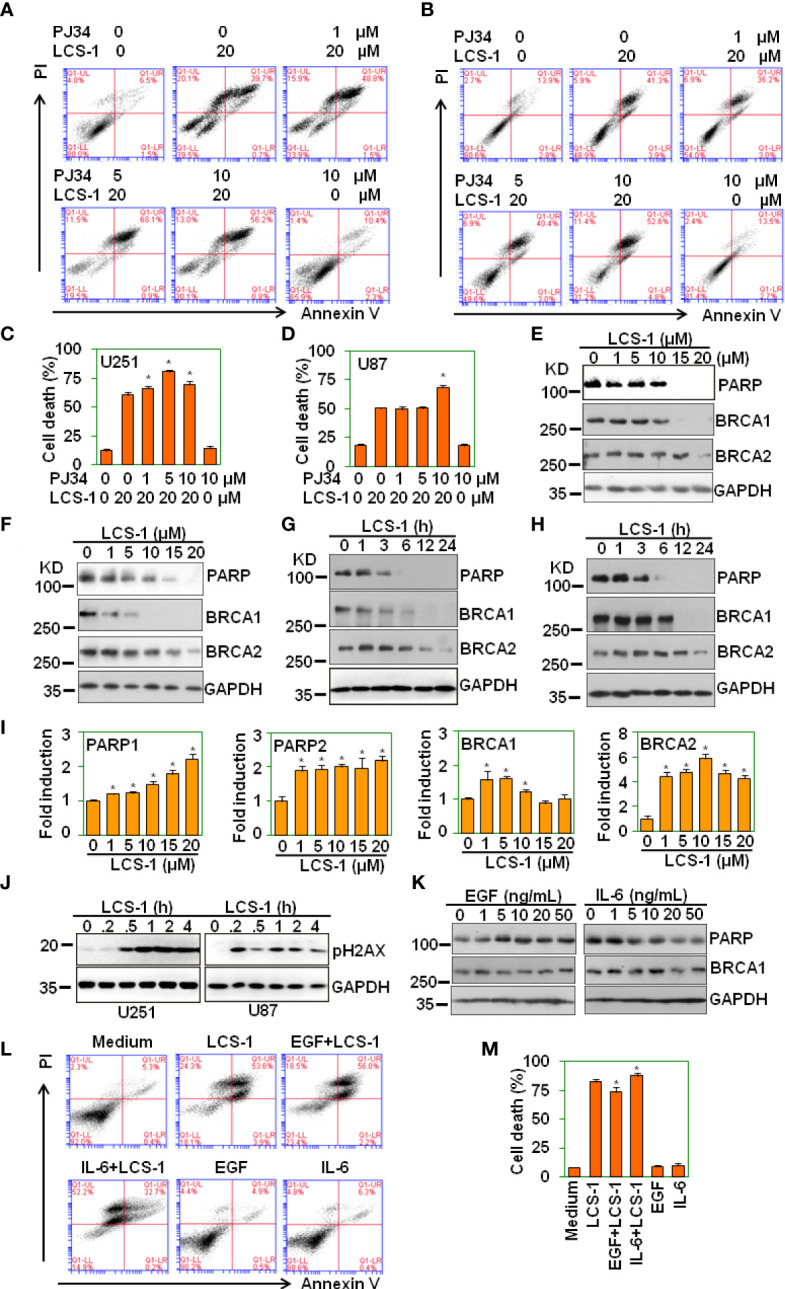
LCS-1 induces degradation of both PARP and BRCA1. **(A)** PI/FITC-Annexin V staining of death of U251 glioma cells pretreated with the indicated doses of PARP inhibitor PJ34 for 1 h. and retreated with 20 μM LCS-1 for 24 h **(B)** PI/FITC-Annexin V staining of death of U87 glioma cells pretreated with the indicated doses of PARP inhibitor PJ34 for 1 h. and retreated with 20 μM LCS-1 for 24 h. **(C, D)** Quantitative data of **A (C)** and B **(D)** respectively. * *P* < 0.05 compared with LCS-1-treated alone groups. **(E, F)** Western blot analysis of the protein levels of PARP, BRCA1 and BRCA2 in U251 **(E)** and U87 **(F)** cells treated with the indicated doses of LCS-1 for 24 h. GAPDH protein levels were measured as loading controls. **(G, H)** Western blot analysis of the protein levels of PARP, BRCA1 and BRCA2 in U251 **(G)** and U87 **(H)** cells treated with 20 μM LCS-1 for the indicated time periods. GAPDH protein levels were measured as loading controls. **(I)** qRT-PCR analysis of the mRNA levels of PARP1, PARP2, BRCA1 and BRCA2 in U251 cells treated with the indicated doses of LCS-1 for 12 h. * *P* < 0.05 compared with the control groups. **(J)** Western blot analysis of the phosphorylated levels of H2AX in U251 and U87 cells treated with 20 μM LCS-1 for the indicated time periods. GAPDH protein levels were measured as loading controls. **(K)** Western blot analysis of the protein levels of PARP, BRCA1 in U87 cells treated with the indicated doses of EGF, or IL-6 for 24 h. GAPDH protein levels were measured as loading controls. **(L)** PI/FITC-Annexin V staining of death of U87 glioma cells pretreated with 20 ng/mL EGF, or 20 ng/mL IL-6 for 24 h. and retreated with 20 μM LCS-1 for 24 h. **(M)** Quantitative data from L. * *P* < 0.05 compared with LCS-1-treated alone group.

Parthanatos is a poly(ADP-ribose) polymerase (PARP)-dependent programed cell death (29, 30), and ROS may elicit Parthanatos (31). To determine LCS-1-induced cell death is associated with parthanatos, U251 and U87 cells were pretreated with PARP-1 inhibitor PJ34, and retreated with LCS-1, and cell death was measured. The results showed that PJ34 did not reverse LCS-1-induced cell death (**Figures 9A-D**). On the contrary, PJ34 treatment increased LCS-1-induced cell death slightly but significantly (**Figures 9A-D**). Considering that PARP inhibitors can elicit cell death in BRCA1 or BRCA2 mutant breast cancer cells (32), we speculated that LCS-1 may induce cell death *via* degrading PARP and BRCA1. This hypothesis was confirmed by the observation that LCS-1 treatment induced the degradation of PARP and BRCA1 dose- and time-dependent in both U251 and U87 cells (**Figures 9E-H**). However, LCS-1 did not induce the decrease of the mRNA levels of PARP and BRCA1 **(** **Figure 9I** **)**. Meanwhile, LCS-1 treatment increased the phosphorylated levels of H2AX, which is a maker for DNA damage **(** **Figure 9J** **)**. Moreover, EGF increased the expression of PARP, but IL-6 decreased the expression of PARP **(** **Figure 9K** **)**. Expectedly, EGF decreased the cell death induced by LCS-1, and IL-6 increased cell death induced by LCS-1 **(** **Figures 9L, M** **)**. These results suggest that LCS-1-induced cell death is not associated with parthanatos, but may be associated with the degradation of PARP and BRCA1.

## Discussion

In normal cells, SOD1 localizes in the cytoplasm, the inter-membrane space of the mitochondria and the nucleus (33). Observed evidences from several groups indicate that SOD1 is upregulated in cancers and is essential to maintain cellular redox balance under the condition with excessive ROS derived from the aberrant metabolism (33, 34). The SOD activities in normal and tumor breast tissues are determined, and each donor has a higher SOD activity in cancer than in normal tissue samples (35). In cisplatin resistant human ovarian cancer cells, the SOD1 expression is higher than that in cisplatin-sensitive human ovarian cancer cells (36). In breast cancer, no difference is found in SOD1 levels between matched plasma and nipple aspirate fluid (NAF) from cancer patients, whereas SOD1 levels in no-cancer NAF are significantly higher compared with matched plasma (37). In lung cancer patients, erythrocyte SOD1 activities are significantly higher than those in normal controls (38). In bronchial epithelium adjacent to invasive cancer, the expression of cytoplasmic or nuclear SOD1 is significantly lower compared with its expression in the uninvolved bronchial epithelium away from cancer (39). In breast cancer cells, SOD2 to SOD1 switch is found, resulting in the SOD2 down-regulation, and SOD1 upregulation, and SOD1 functions to maintain the integrity of the organelle (16). A significant upregulation of SOD1 in nasopharyngeal carcinoma (NPC) tissue is observed and high SOD1 expression is a predictor of poor prognosis and is correlated with poor outcome, indicating that SOD1 is a potential prognostic biomarker and a promising target for NPC therapy (40). However, less evidence is reported about the expression of SOD1 in gliomas. In this study, SOD1 moderate staining was observed in normal brain tissues, glioma adjacent tissues, glioma grade I and II tissues, whereas SOD1 strong staining was found in glioma grade III, IV tissues. The higher expression of SOD1 in glioma tissue may be due to the higher levels of ROS, which are derived from the aberrant metabolism. In glioma cell lines, SOD1 inhibitor induced ROS production, activated ROS signaling, and increased SOD1 expression. So we consider that the upregulation of SOD1 in glioma may be associated with the high levels of ROS.

SOD function to catalyze superoxide anion into oxygen and hydrogen peroxide, to decrease ROS levels, to maintain cellular redox homeostasis. SOD dysfunction leads to excessive increase of ROS, blocks redox balance, and results in tissue and cell damage. However, in cancer cells, the cell damage induced by SOD dysfunction should benefit to cancer therapy. Early evidences show that SOD1 inhibitor ATN-224 inhibits endothelial cell proliferation *in vitro*, and attenuates angiogenesis *in vivo* (12). The effects of ATN-224 on endothelial and tumor cells could be substantially reversed using a catalytic small-molecule SOD mimetic (12). Other evidences show that inhibition of SOD1 by ATN-224 induces cell death in various non–small-cell lung cancer (NSCLC) cells, including those harboring KRAS mutations (13). ATN-224 inhibition of SOD1 increases superoxide, which diminishes enzyme activity of the antioxidant glutathione peroxidase, leading to an increase in intracellular hydrogen peroxide levels (13). By combining affinity proteomics and gene expression analysis, a small molecule, referred to as lung cancer screen 1 (LCS-1) is identified as SOD1 inhibitor and reduces the growth of lung adenocarcinoma cell lines (14). Overexpression of SOD1 increases proliferation of lung cancer cells and reduces sensitivity of these cells to LCS-1 (14). Chebulinic acid (CA), a polyphenol derived from the fruits of various medicinal plants, downregulates the expression of SOD1, reduces its enzyme activity, elicits cell oxidative stress, inhibits cell proliferation and promotes cell apoptosis in breast cancer cells (41). In this study, SOD1 inhibitor LCS-1 induced time- and dose-dependent cell death in glioma cells. And LCS-1 reduced growth of glioma *in vivo*. These observations suggest that targeting SOD1 may be a strategy for glioma therapy.

Several natural or synthetical compounds have been reported to induce anti-glioma effect *via* ROS-dependent mechanism. WIN 55,212-2, a cannabinoid analogue, dose-dependently inhibits glioma cell proliferation, migration, and invasion *in vitro*, effectively suppresses glioma spheroids growth *ex vivo* (42). WIN 55,212-2 also induces significant apoptosis, and causes dysfunction of VEGF-AKT/FAK signaling (42). The effects of WIN 55,212-2 are ROS-dependent, ROS inhibition effectively attenuates dysfunction of VEGF-AKT/FAK signaling and eventually improves glioma cell proliferation, migration, and invasion (42). Osthole, a coumarin derivative, is found to trigger glioma cell necroptosis accompanied with ROS production (43).. Osthole treatment decreases the expression of necroptosis inhibitor caspase-8, and the levels of necroptosis proteins receptor-interacting protein 1 (RIP1), RIP3 and mixed lineage kinase domain-like protein (43). The pretreatment with RIP1 inhibitor necrostatin-1 attenuates both osthole-induced necroptosis and the production of ROS in glioma cells (43). Natural borneol has been reported to sensitize human glioma cells to cisplatin-induced apoptosis by triggering ROS-mediated oxidative damage and regulating MAPK and PI3K/AKT signaling (44). Paris polyphyllins are monomers extracted from rhizome of Paris polyphylla var. yunnanensis. Polyphyllin VII promotes apoptosis and autophagic cell death *via* ROS-inhibited AKT activity, and sensitizes glioma cells to temozolomide (45). Ampelopsin, an effective component of the traditional Chinese herb of Ampelopsis grossedentata, inhibits human glioma through inducing apoptosis and autophagy dependent on ROS generation and JNK pathway (46). Fucoxanthin, a natural carotenoid derived from algae, induces apoptosis in human glioma cells *via* triggering of ROS-mediated oxidative damage and regulation of MAPKs and PI3K-AKT pathways (47). In this study, LCS-1 induces ROS production, activates ROS signaling. ROS scavengers reversed LCS-1-induced cell death. These results suggest that LCS-1 induced cell death *via* ROS-dependent pathway.

Multiple evidences show that ROS induced by various factors elicit cell differentiation, cell death, and inhibit tumor growth *via* P53 pathway (24–26). In this study, we found that LCS-1 has less effect on P53-targeted genes, indicating that LCS-1 did not activate P53. Meanwhile, P53 inhibitor did not reverse LCS-1-induced cell death, suggesting that LCS-1-induced cell death is P53-independent.

ROS have been reported to induce tissue damage, to elicit anti-tumor immune response, to cause cell death *via* caspase 1, 3 and 8 pathways (27, 28). However, in this study, we found that LCS-1 did not activate caspase 3. Meanwhile, caspase pan-inhibitor did not reverse LCS-1-induced cell death. These results suggest that LCS-1-induced cell death is caspase-independent.

PARP has been reported to involve in inflammatory response and cell death induced by ROS (48). In this study, we found that SOD1 inhibitor LCS-1 induced ROS-dependent cell death. But PARP inhibitor did not reverse LCS-1-induced cell death, suggesting that LCS-1-induced cell death is not asssociated with PARP activation.

ROS induces DNA damage and activates DNA damage responses (49). There are three ways to repair DNA damage: the PARylation-mediated repair, the homologous recombination (HR)-mediated repair, and end-joining (EJ)-mediated repair (32). Upon DNA damage, PARP is rapidly recruited to single-strand breaks (SSBs) and double-strand breaks (DSBs) where it PARylates itself and other proteins resulting in the recruitment of downstream DNA repair factors (32). In BRCA-proficient cells, HR enables the error-free repair of DNA damage (32). By contrast, BRCA1/2-deficient cells are HR-deficient and are therefore reliant upon error-prone DNA end-joining pathway, in which PARP is necessary (32, 50). Therefore, the treatment of BRCA1/2-deficient cells with PARP inhibitors blocks all three DNA damage repair pathways, leading to the induction of cell death, and these inhibitors have been used as cancer therapeutic strategies (51–53).

**Figure 10:**
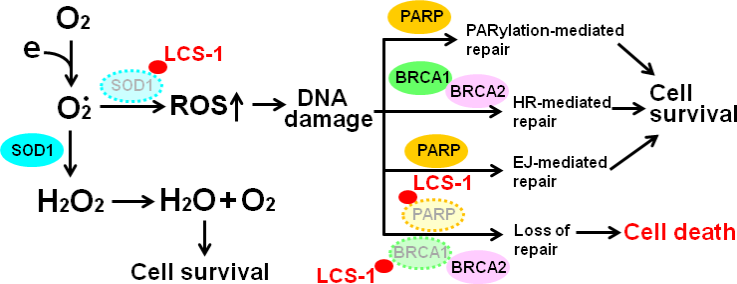
Model of the mechanism by which LCS-1 induces cell death. LCS-1 inhibited the enzyme activity of SOD1, resulting in the accumulation of ROS, leading to the induction of DNA damage. Meanwhile, LCS-1 induces the degradation of PARP. The dysfunction of PARP inhibits DNA damage repair *via* blocking both PARylation-mediated and EJ-mediated pathways. Furthermore, LCS-1 induces the degradation of BRCA1, eliciting the block of HR-mediated pathway. The inhibition of these three repair pathways results in death of glioma cells.

The results presented in this study are compatible with the model outlined in **Figure 10**. The treatment of glioma cells with LCS-1 inhibits SOD1, resulting in the accumulation of ROS, leading to DNA damage. Meanwhile, LCS-1 induces the degradation of PARP, which cause the dysfunction of PARylation-mediated repair and EJ-mediated repair, and the degradation of BRCA1, which causes the blocking of HR-mediated repair. Combining these effects of LCS-1 in glioma cells, it induces death of glioma cells through the similar mechanism compared to that PARP inhibitors induce cell death in BRCA1/2 deficient cells.

## Data availability statement

The raw data supporting the conclusions of this article will be made available by the authors, without undue reservation.

## Ethics statement

The studies involving human participants were reviewed and approved by the ethics committee of Changsha Central Hospital, University of South China. The patients/participants provided their written informed consent to participate in this study. The animal study was reviewed and approved by the Animal Ethics Committee of the Changsha Central Hospital, University of South China.

## Author contributions

JH conceived and designed the study. ML, QL, YW, XL, MJ, and JH collected and analyzed the data.

ML and QL completed the experimental cell manipulation. YW completed immunohistochemistry of SOD1 and immunohistochemical scoring. ML and XL completed western blot analysis. ML and MJ completed qRT-PCR analysis. JH wrote the manuscript. All authors revised the manuscript and read and approved the submitted version.

## Funding

This study was supported by the National Natural Science Foundation of China (81172042).

## Conflict of interest

The authors declare that the research was conducted in the absence of any commercial or financial relationships that could be construed as a potential conflict of interest.

## Publisher’s note

All claims expressed in this article are solely those of the authors and do not necessarily represent those of their affiliated organizations, or those of the publisher, the editors and the reviewers. Any product that may be evaluated in this article, or claim that may be made by its manufacturer, is not guaranteed or endorsed by the publisher.

## Abbreviations

BRCA1, breast cancer gene 1; EJ, end-joining; FITC, fluorescein isothiocyanate; GAPDH, glyceraldehyde-3-phosphate dehydrogenase; GCLC, γ-glutamyl cysteine ligase catalytic subunits; GCLM, γ-glutamyl cysteine ligase modulatory subunits; GSH, reduced glutathione; HO-1, heme oxygenase-1; HR, homologous recombination; IHC, immunohistochemistry; LCS-1, lung cancer screen 1; NAC, N-acetyl-L-cysteine; NPC, nasopharyngeal carcinoma; NQO1, NAD(P)H dehydrogenase quinone 1; NSCLC, non-small-cell lung cancer; PARP, poly (ADP-ribose) polymerase; PI, propidium iodide; qRT-PCR, quantitative reverse transcriptional-polymerase chain reaction; RIP1, receptor-interacting protein 1; ROS, Reactive oxygen species; SOD1, superoxide dismutase 1.
