# Supplementary material for: LCS-1 inhibition of superoxide dismutase 1 induces ROS-dependent death of glioma cells and degradates PARP and BRCA1
Source: Front Oncol. 2022 Aug 1;12:937444. doi: 10.3389/fonc.2022.937444 (PMC9376264; doi:10.3389/fonc.2022.937444)

## Slide 1
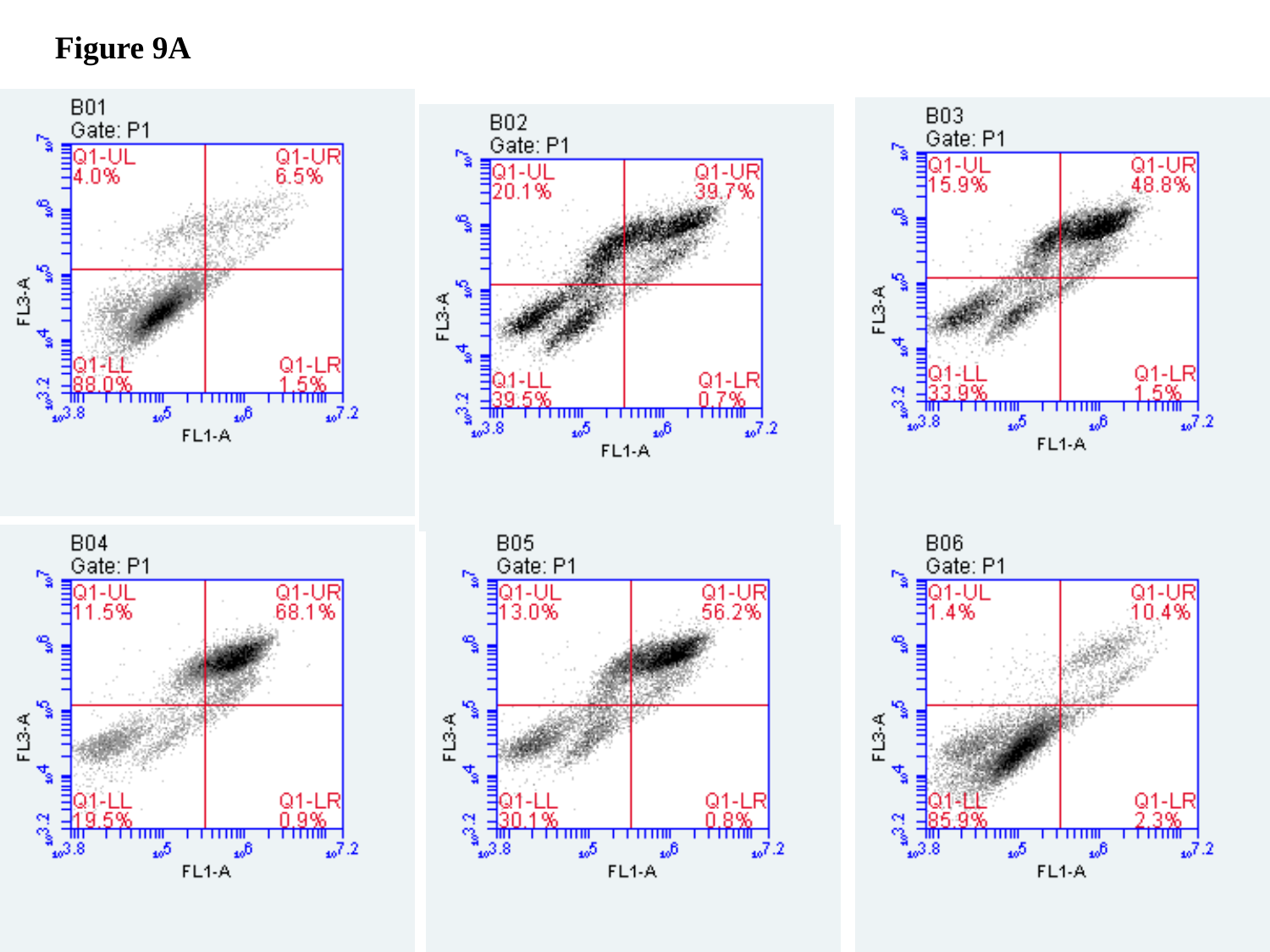

Figure 9A

## Slide 2
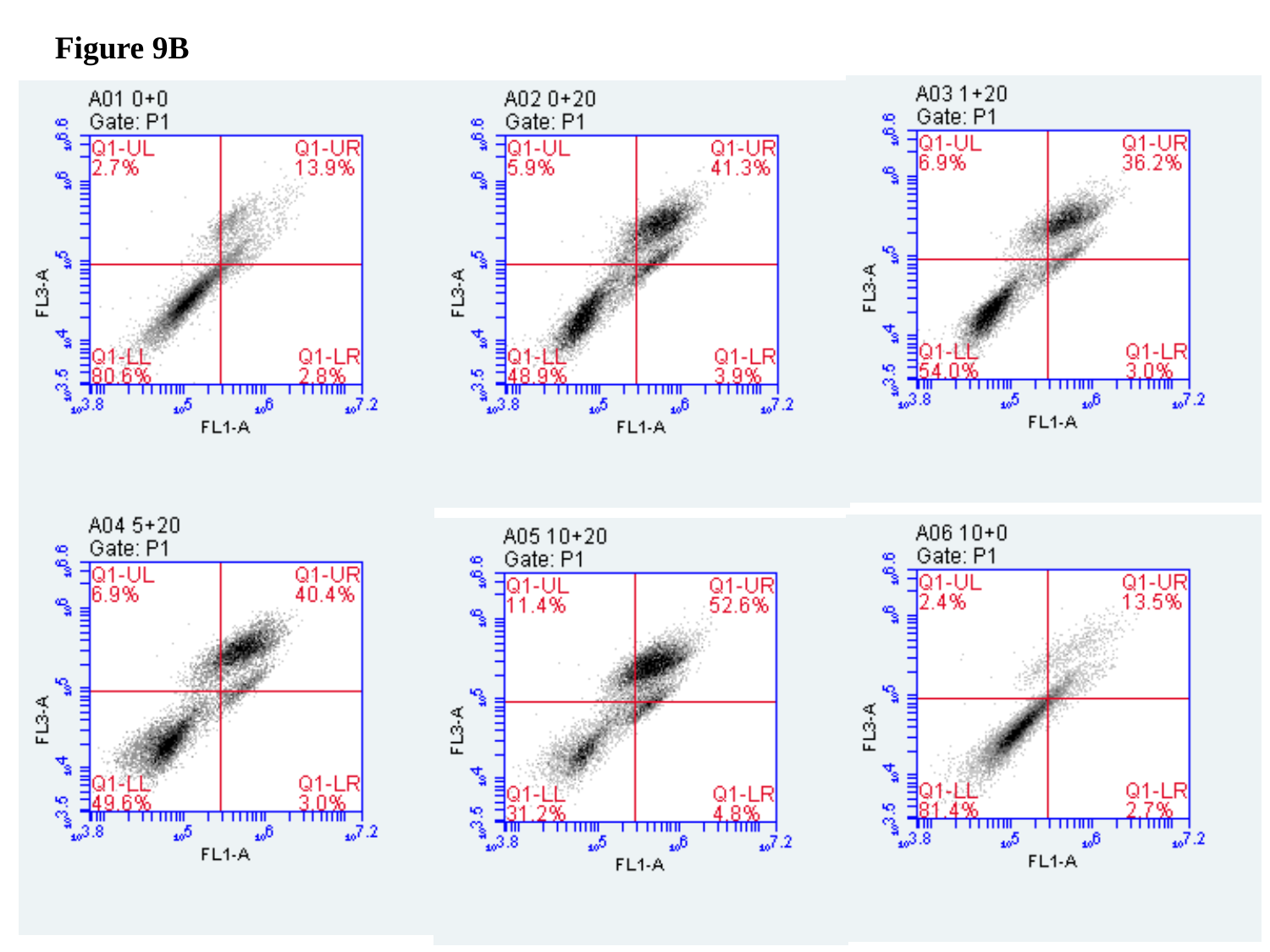

Figure 9B

## Slide 3
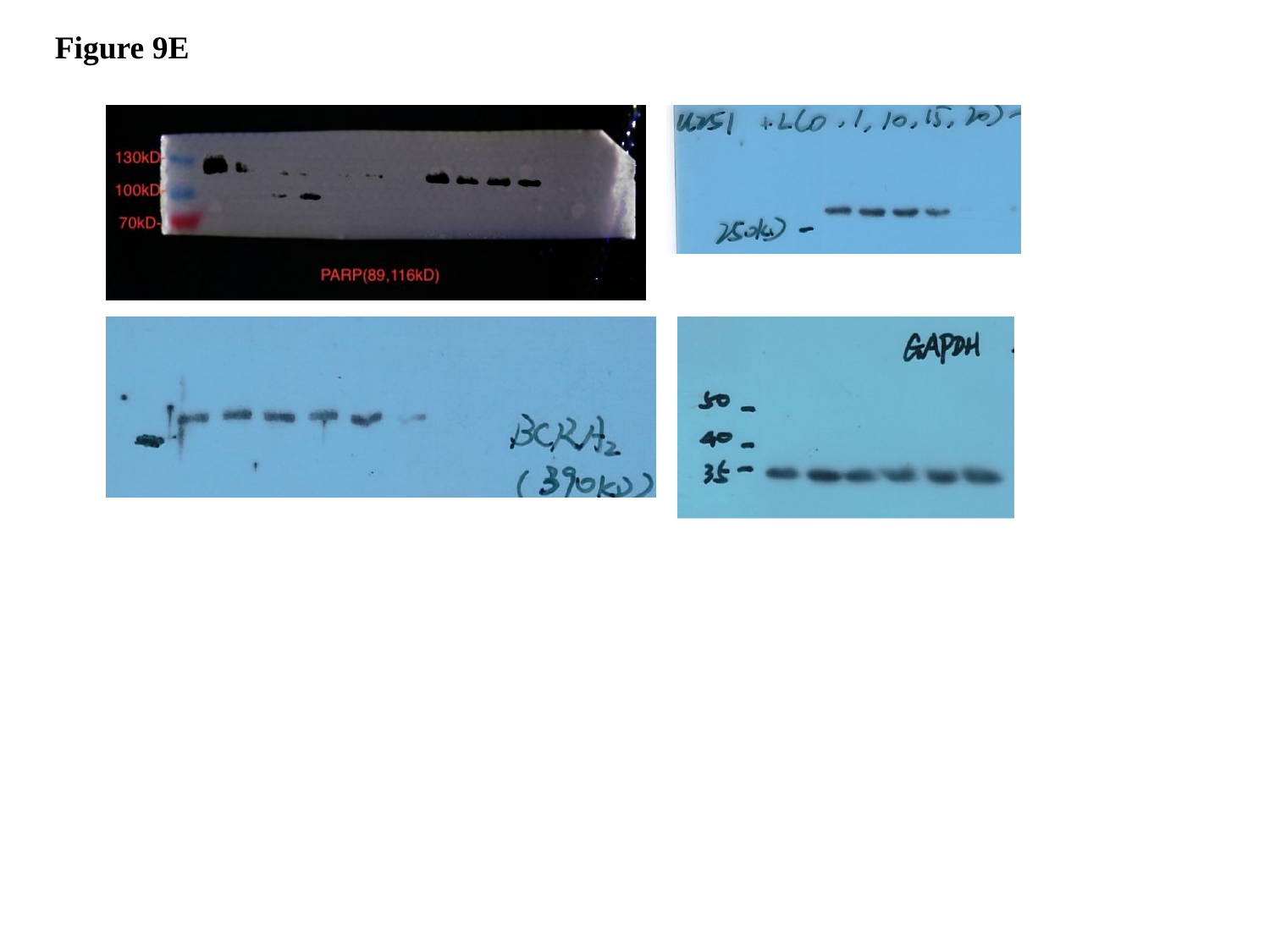

Figure 9E

## Slide 4
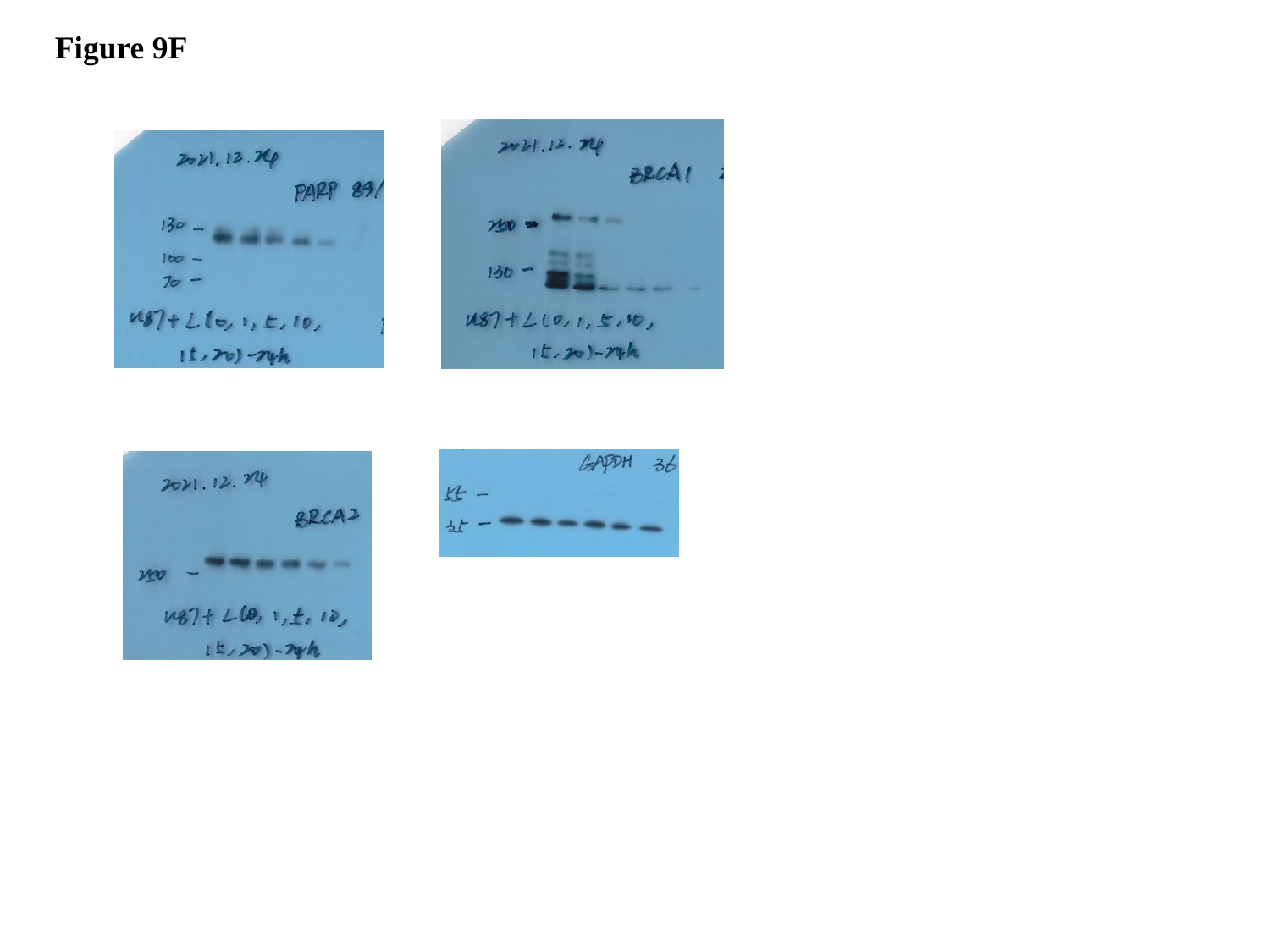

Figure 9F

## Slide 5
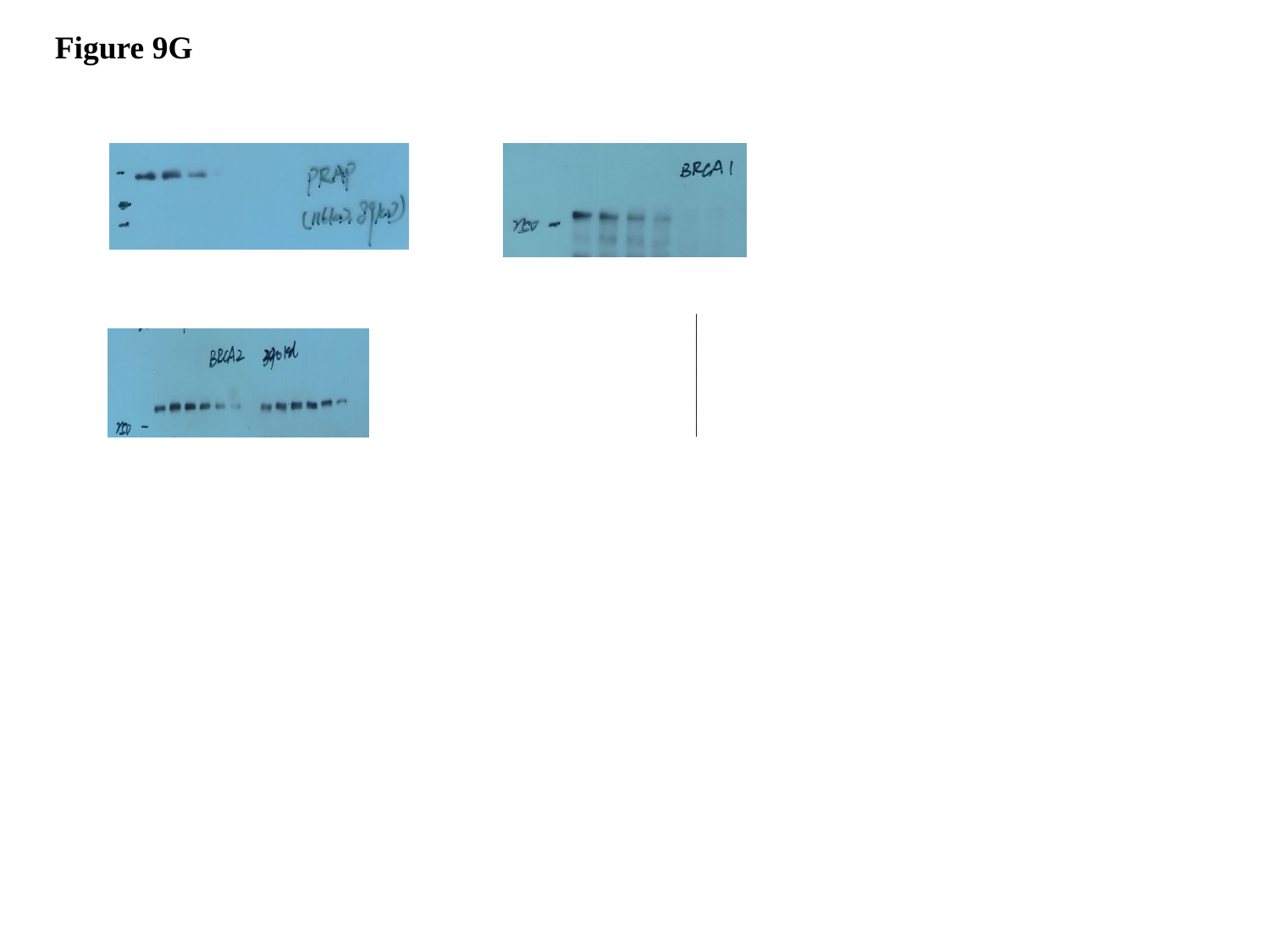

Figure 9G

## Slide 6
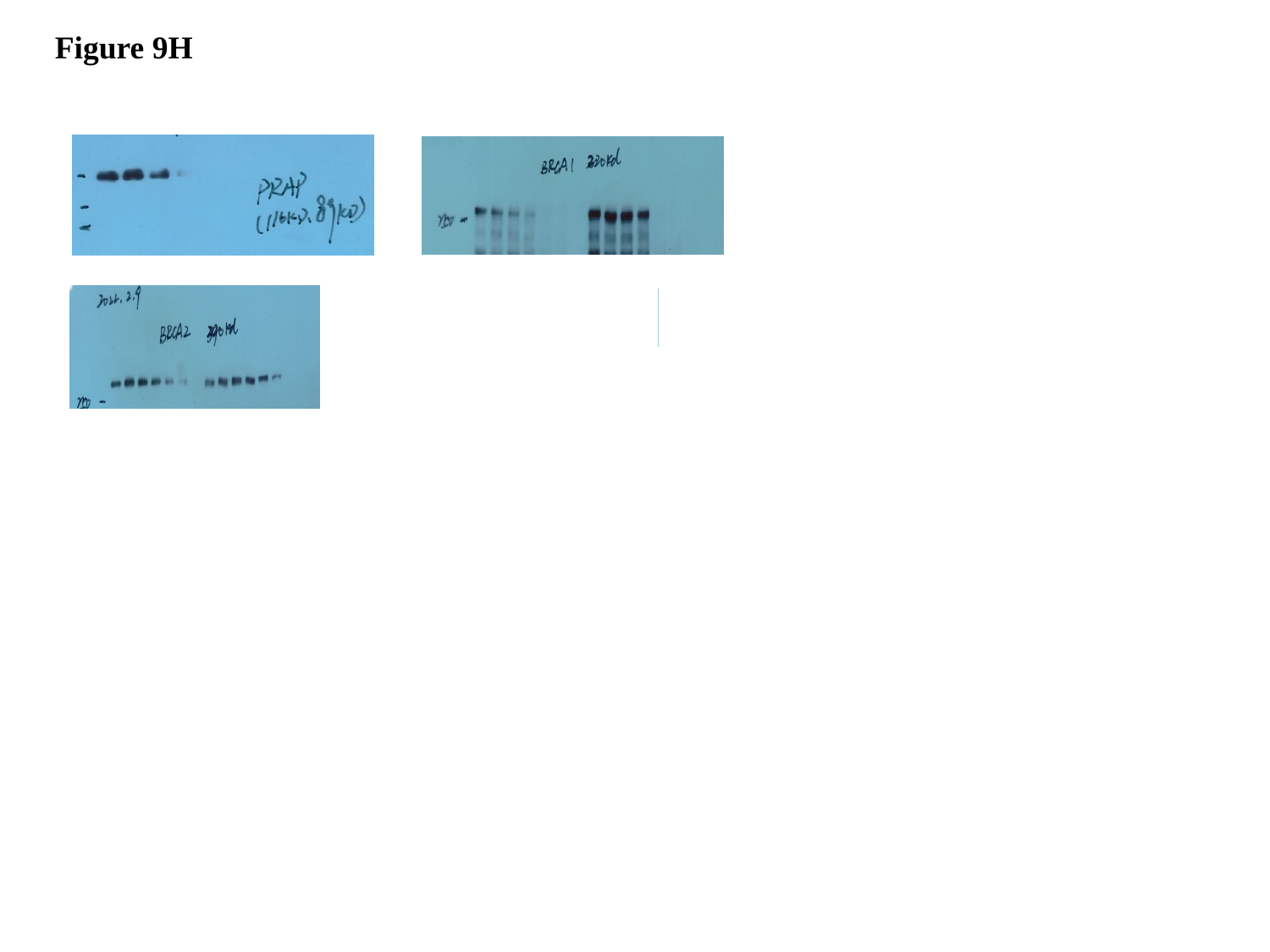

Figure 9H

## Slide 7
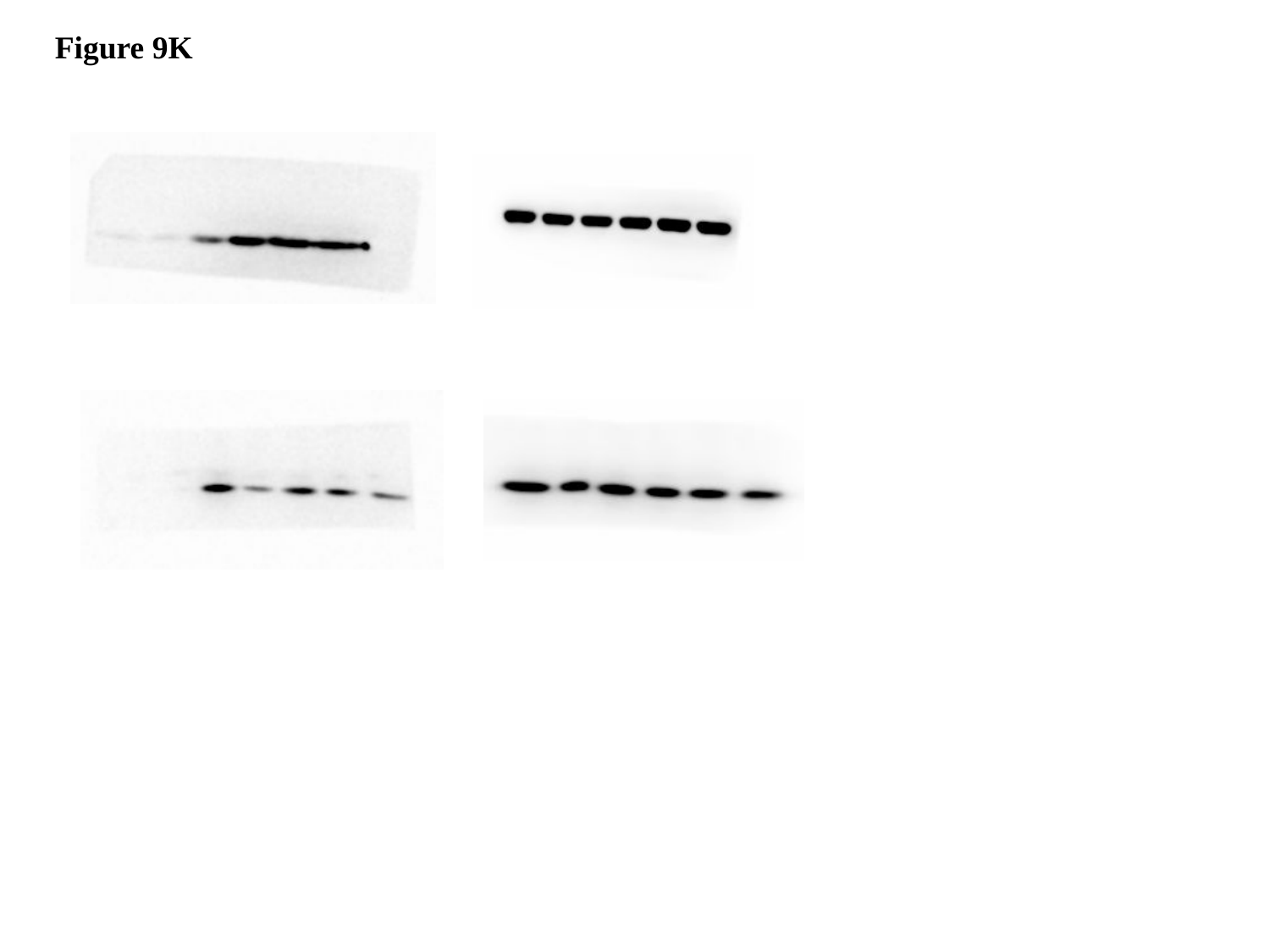

Figure 9K

## Slide 8
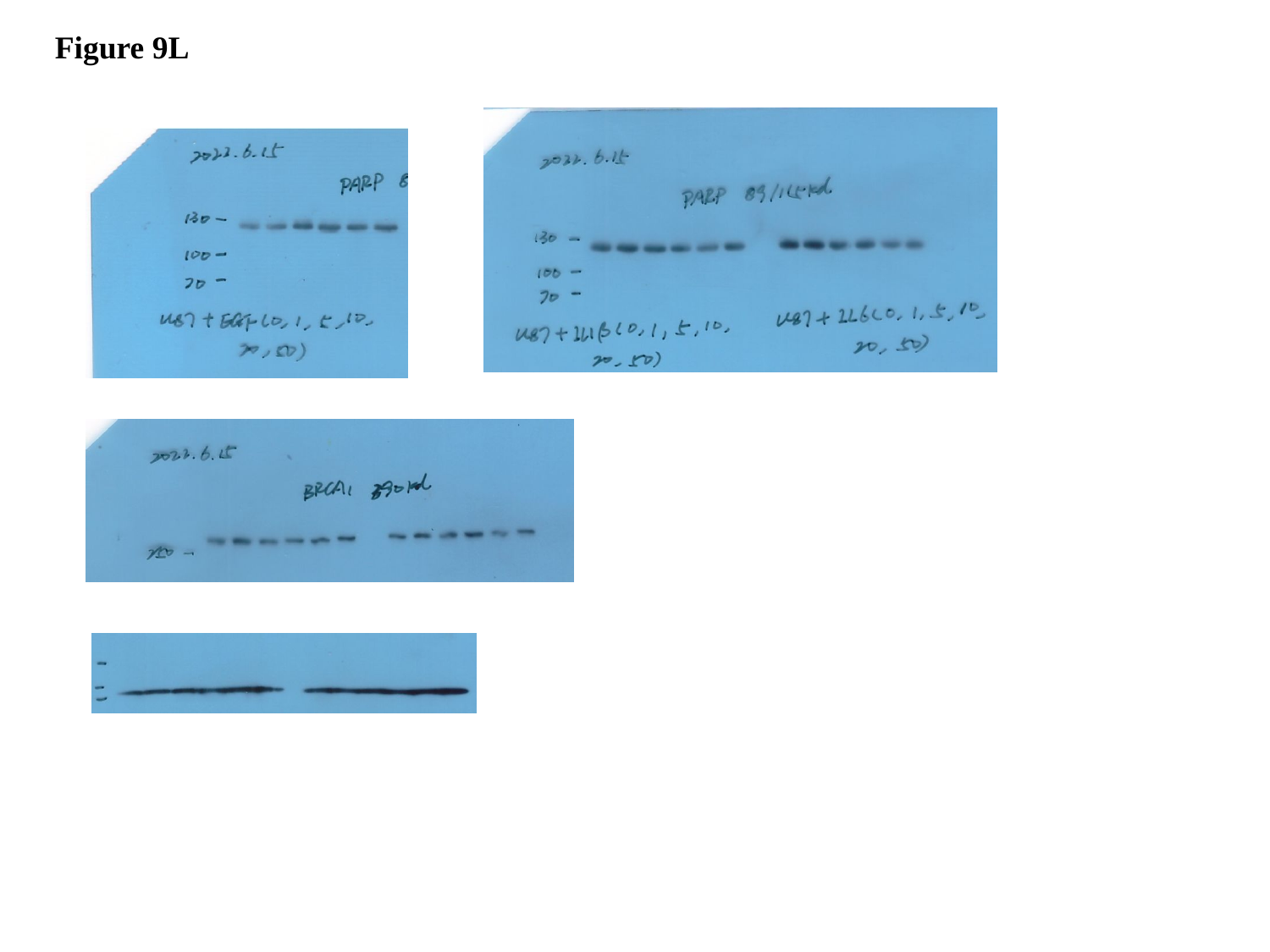

Figure 9L

## Slide 9
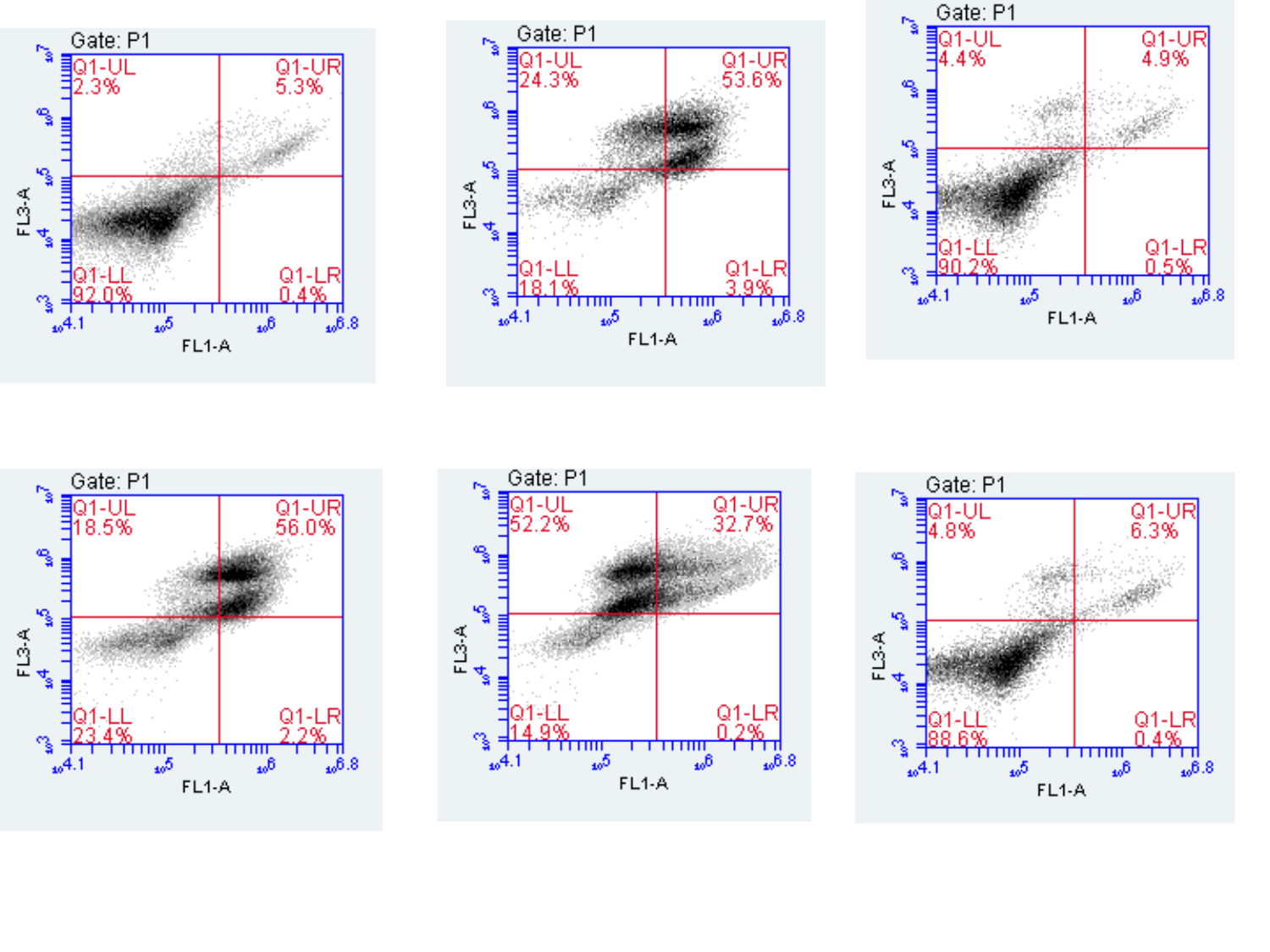

Supplement: Supplementary file 2 [file Presentation_1.pptx]
